# Supplementary material for: Multi-Omic Analysis Reveals Different Effects of Sulforaphane on the Microbiome and Metabolome in Old Compared to Young Mice
Source: Microorganisms. 2020 Sep 29;8(10):1500. doi: 10.3390/microorganisms8101500 (PMC7599699; doi:10.3390/microorganisms8101500)
Supplement: Supplementary file 1 [file microorganisms-08-01500-s001.pdf]

Supplementary Materials:

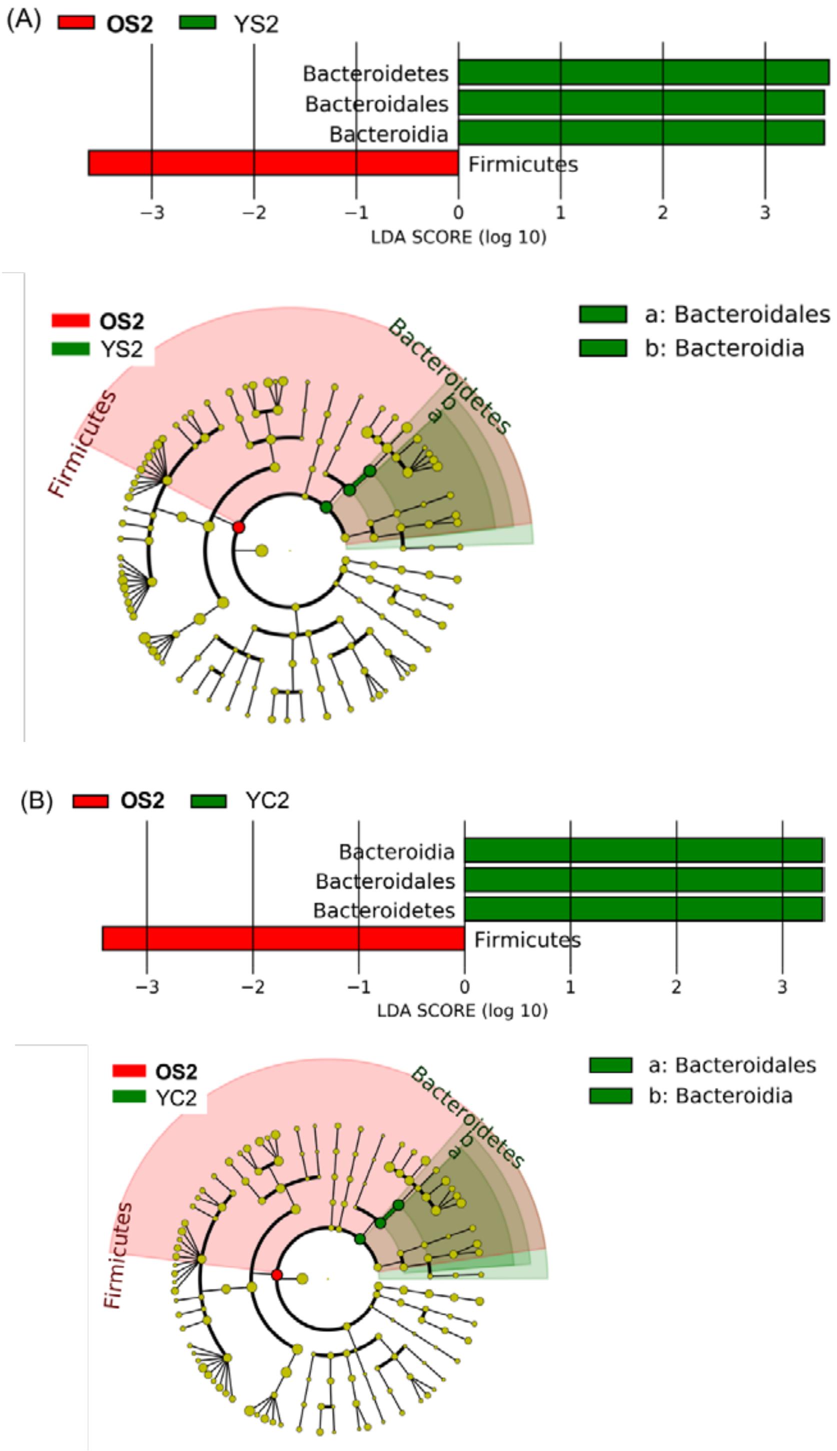

Figure S1. Identification of bacterial biomarkers using linear discriminant effect size analysis. (A) Comparison of old SFN-treated mice (**OS2**) with young SFN-treated mice (YS2). (B) Comparison of **OS2** and young mice that received control diet for 2 months (YC2). The distribution bar chart of LDA values shows the species with LDA scores greater than 3 and the species with significantly different abundances in different groups. The length of the histogram represents the size of the impact of significantly different species. The circle radiating from inside to outside represents the classification from the phylum to the genus level. Each small circle represents a classification at that level at different classification levels. Those taxa in each level are colored by farm for which it is more abundant. The diameter of the small circle is proportional to the relative abundance. Note that no differentially abundant species between YC2 and YS2 were identified.

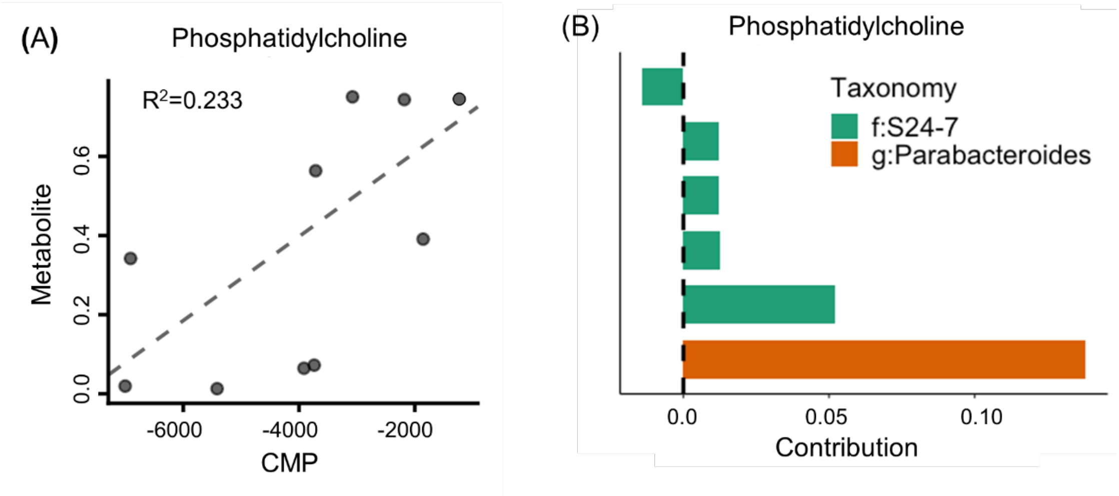

Figure S2. MIMOSA analysis results. (A) MIMOSA identified Phosphatidylcholine (C00157) as well-predicted with a model p-value < 0.1 and a positive model slope. The scatter plot indicates the relationship between experimentally measured values and community-wide metabolic potential (CMP) for each sample for Phosphatidylcholine. (B) MIMOSA identified 29 organisms contributing to Phosphatidylcholine, which were narrowed down to 6 organisms based on abundance, sample frequency and contribution. Contribution bar plots are colored by the lowest taxonomy rank.

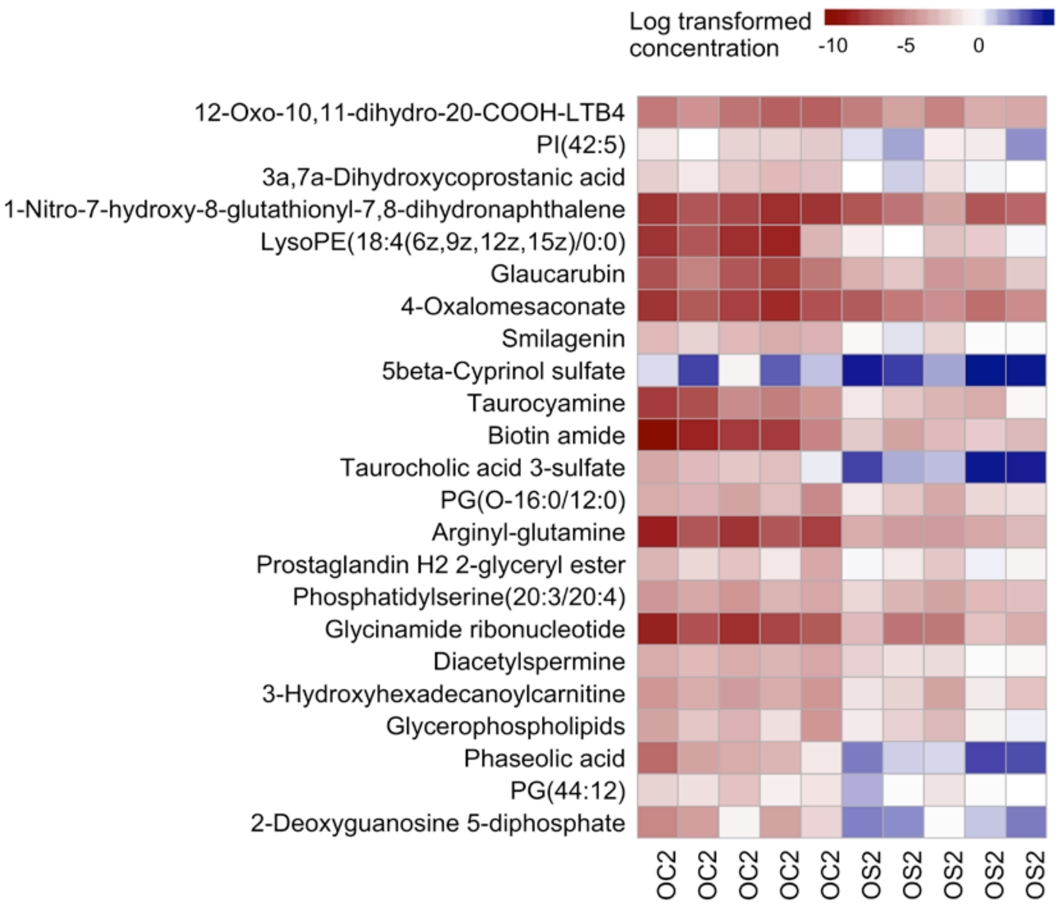

Figure S3. The log-transformed concentration profiles of well-predicted metabolites by MelonnPan in **Figure 6**.

Table S1. Alpha diversity significance test. The Kruskal-Wallis test was used to calculate p-value between experimental groups for phylogenetic diversity index and species richness. A p-value <0.05 is flagged with an asterisk.

		P-value for Phylogenetic Diversity Index	P-value for Species Richness
OC0	OC2	0.175	0.117
OC0	OS0	0.251	0.251
OC0	**OS2**	0.917	0.602
OC0	YC0	0.009*	0.059
OC0	YC2	0.347	0.754
OC0	YS0	0.009*	0.175
OC0	YS2	0.754	0.347
OC2	OS0	0.754	0.347
OC2	**OS2**	0.602	0.754
OC2	YC0	0.009*	0.009*
OC2	YC2	0.009*	0.016*
OC2	YS0	0.009*	0.009*
OC2	YS2	0.076	0.175
OS0	**OS2**	0.754	0.917
OS0	YC0	0.009*	0.009*
OS0	YC2	0.028*	0.142
OS0	YS0	0.009*	0.009*
OS0	YS2	0.465	0.602
**OS2**	YC0	0.175	0.175
**OS2**	YC2	0.602	0.754
**OS2**	YS0	0.465	0.465
**OS2**	YS2	0.917	0.754
YC0	YC2	0.009*	0.009*
YC0	YS0	0.175	0.175
YC0	YS2	0.076	0.009*
YC2	YS0	0.016*	0.016*
YC2	YS2	0.117	0.175
YS0	YS2	0.076	0.028*

Table S2. Beta-diversity significance test. The p-values of significant differences in beta diversity between experimental groups were calculated using permutational multivariate analysis of variance (PERMANOVA) with 999 Monte Carlo permutations. A p-value <0.05 is flagged with an asterisk.

		Bray Curtis	unweighted UniFrac	weighted UniFrac	Jaccard
OC0	OC2	0.008*	0.014*	0.272	0.006*
OC0	OS0	0.040*	0.020*	0.184	0.021*
OC0	**OS2**	0.010*	0.006*	0.009*	0.007*
OC0	YC0	0.009*	0.011*	0.045*	0.007*
OC0	YC2	0.009*	0.006*	0.007*	0.009*
OC0	YS0	0.010*	0.008*	0.096	0.010*
OC0	YS2	0.006*	0.010*	0.040*	0.009*
OC2	OS0	0.006*	0.006*	0.276	0.006*
OC2	**OS2**	0.019*	0.164	0.004*	0.110
OC2	YC0	0.011*	0.009*	0.027*	0.005*
OC2	YC2	0.010*	0.012*	0.017*	0.008*
OC2	YS0	0.006*	0.009*	0.035*	0.009*
OC2	YS2	0.115	0.079	0.033*	0.056
OS0	**OS2**	0.009*	0.006*	0.009*	0.012*
OS0	YC0	0.010*	0.008*	0.009*	0.007*
OS0	YC2	0.012*	0.013*	0.020*	0.005*
OS0	YS0	0.017*	0.006*	0.035*	0.010*
OS0	YS2	0.005*	0.007*	0.030*	0.008*
**OS2**	YC0	0.010*	0.012*	0.007*	0.008*
**OS2**	YC2	0.008*	0.007*	0.006*	0.011*
**OS2**	YS0	0.008*	0.007*	0.007*	0.010*
**OS2**	YS2	0.054	0.158	0.005*	0.102
YC0	YC2	0.007*	0.009*	0.010*	0.004*
YC0	YS0	0.063	0.025*	0.164	0.063
YC0	YS2	0.007*	0.010*	0.016*	0.012*
YC2	YS0	0.018*	0.006*	0.009*	0.008*
YC2	YS2	0.107	0.006*	0.585	0.012*
YS0	YS2	0.018*	0.012*	0.007*	0.012*

Table S3. The list of 61 metabolite biomarkers between OS2 and OC2 identified by MelonnPan.

Metabolite	Description
3-Carbamoyl-2-phenylpropionic acid	Drug
Lisuride	Drug
Tazarotene	Drug
SCHEMBL4549604	Drug
Clopidogrel	Drug
Ergonovine	Drug
Atpenin A5	Drug
ortho-hydroxyrosiglitazone	Drug
SCHEMBL21067798	Drug
SCHEMBL4316970	Drug
Ambenonium	Drug
Dirithromycin	Drug
Ethosuximide	Drug
Repaglinide aromatic amine	Drug
Vardenafil	Drug
Hydroxyritonavir	Drug
Traumatic acid	Plant
N-jasmonoyltyrosine	Plant
Nigakihemiacetal B	Plant
Divanillyltetrahydrofuran ferulate	Plant
Proscillaridin A	Plant
Simmondsin 2'-ferulate	Plant
Acrimarine J	Plant
25-Acetyl-6,7-didehydrofevicordin F 3-glucoside	Plant
Koenimbine	Plant
Prostaglandin H2 2-glyceryl ester	Lipid
Smilagenin	Lipid
Glaucarubin	Lipid
5beta-Cyprinol sulfate	Bile acid biosynthesis

3a,7a-Dihydroxycoprostanic acid	Bile acid
Biotin amide	Biotin
Glycinamide ribonucleotide	Purine
Arginyl-glutamine	Dipeptide
Ganoderic acid xi	Fungi
4-Oxalomesaconate	Protocatechuate (PCA) 4,5-cleavage (PCA45) pathway
1-Nitro-7-hydroxy-8-glutathionyl-7,8-dihydronaphthalene	Glutathione
Azaspiracid	Marine algal toxin
Taurocyamine	
Cyclic siloxane	
Phaseolic acid	
Vidarabine	
2,4-Ditert-butyl-6-(5-chlorobenzotriazol-2-yl)phenol	
Diacetylspermine	
1XUX	
2'-Deoxyguanosine 5'-diphosphate	
3-Hydroxyhexadecanoylcarnitine	
Pangamic acid	
Eurysterol B sulfonic acid	
Steroid ester	
PS(19:0/0:0)	
PI(17:1(10Z)/0:0)	
Taurocholic acid 3-sulfate	
PG(O-16:0/12:0)	
Glycerophospholipids	
Phosphatidylserine(20:3/20:4)	
PG(44:12)	
TG(57:6)	
3,5-Dinitro-4-hydroxyphenylpyruvate	
12-Oxo-10,11-dihydro-20-COOH-LTB4	

LysoPE(18:4(6z,9z,12z,15z)/0:0)	
PI(42:5)	
